# Blending Modification of Alicyclic Resin and Bisphenol A Epoxy Resin to Enhance Salt Aging Resistance for Composite Core Rods

**DOI:** 10.3390/polym14122394

**Published:** 2022-06-13

**Authors:** Yunpeng Liu, Wanxian Wang, Hechen Liu, Mingjia Zhang, Jie Liu, Junwei Qi

**Affiliations:** 1 Hebei Provincial Key Laboratory of Power Transmission Equipment Security Defence, North China Electric Power University, Yonghua North Street No. 619, Baoding 071003, China; liuyunpeng@ncepu.edu.cn (Y.L.); 220192213050@ncepu.edu.cn (W.W.); 18367683603@163.com (M.Z.); jumwencepu@163.com (J.Q.); 2 State Key Laboratory of Alternate Electrical Power System with Renewable Energy Sources, North China Electric Power University, Yonghua North Street No. 619, Baoding 071003, China; 3 State Grid Hebei Electric Power Company Electric Power Research Institute, Xingan Street No. 200, Yuhua District, Shijiazhuang 050021, China; 18633015914@163.com

**Keywords:** DGEBA, alicyclic epoxy resin, blending modification, salt corrosion resistance, insulation performance

## Abstract

In order to promote the application of composite insulators in coastal areas with high temperature, high humidity and high salt, it is of great importance to develop matrix resin with salt corrosion resistance for composite core rods. In this study, bisphenol A epoxy resin was modified by blending with alicyclic epoxy resin (2021P). Three different proportions of 2021P/DGEBA blend resins (0% 2021P/DGEBA, 10% 2021P/DGEBA and 20% 2021P/DGEBA) were prepared, and the high salt medium corrosion test was carried out. The physicochemical (FTIR, DMA, TGA) and electrical properties (dielectric loss, leakage current and breakdown field strength) of the blend resin before and after aging were tested and analyzed, and the optimal blend proportion was determined. The results showed that after salt aging, the Tg of 0% 2021P/DGEBA decreased to 122.99 °C, while the Tg of 10% 2021P/DGEBA reached 134.89 °C; The leakage current of 0% 2021P/DGEBA increased to 48.994 μA, while that of 10% 2021P/DGEBA only increased to 44.549 μA; The breakdown field strength of 0% 2021P/DGEBA dropped to 40.36 kv/mm, while that of 10% 2021P/DGEBA only dropped to 43.63 kv/mm. The introduction of 2021P enhanced the salt corrosion resistance of the blend resin, which could hinder the penetration, diffusion and erosion of external media (such as Na^+^, Cl^−^, H_2_O, etc.) to the matrix resin. The comprehensive properties of 10% 2021P/DGEBA blend system reached the best, which was better than other blending resins, showing great application potential.

## 1. Introduction

Epoxy resin materials are more and more widely used in line insulation devices (such as composite insulators, composite cross arms and composite poles, etc.) due to their high mechanical strength, light weight, and good insulation properties. However, the coastal areas of China have special climatic conditions such as high temperature, high humidity and high salt. Composite insulators and other products are easily corroded by salt spray when erected in coastal areas and other places, which destroys the epoxy resin matrix in the mandrel, and eventually lead to the decline of the performance of composite insulators [1,2]. Accordingly, it is very important to explore the influence of different resin matrix materials on the aging resistance of salt and to develop the formulation technology of epoxy resin matrix materials with high salt resistance.

Bisphenol A epoxy resin (DGEBA) is one of the most widely used epoxy resins in various industrial fields because of its excellent thermal properties, electrical insulation properties and physical and mechanical properties. A large number of studies on toughening and weather resistance have been conducted in order to better adapt to various industrial applications [3,4,5,6]. At present, the resin matrix in the composite insulating mandrel mostly adopts bisphenol A epoxy resin, and there have been many studies on the weather resistance modification of bisphenol A epoxy resin. The main methods include the following three aspects: (i) Molecular chemical modification of epoxy resin. Bifulco et al. [7] used melamine, silica nanoparticles and phosphorus-based compounds to improve the fire resistance of bisphenol A epoxy resin. The research results showed that the modified intumescent system achieved significant reduction in heat release rate and delay of the ignition time. (ii) Nano filler modified epoxy resin. Awad [8] et al. respectively added microcrystalline cellulose (MCC) to bisphenol A epoxy resin and corresponding aliphatic epoxy resin for modification, and studied the accelerated weathering of the resin. The results showed that the addition of MCC can improve the thermal stability of epoxy resin, reduce surface oxidation and better retain mechanical properties. In addition, Awad [9] et al. also studied the improvement of aging performance of bisphenol A epoxy resin and its corresponding alicyclic resin by adding calcium sulfate as a photostabilizer. The results showed that adding calcium sulfate to the resin could improve the initial mechanical properties and maintain good properties under simulated accelerated weathering conditions. (iii) Blending modification of epoxy resin and weathering resin. Jiao et al. [10] successfully synthesized the UV-curable urethane acrylate oligomers modified with cycloaliphatic epoxide resin, and the result revealed that the alicyclic structure in the oligomer molecular make-up can effectively improve the adhesion and boiling water resistance of UV-curable coatings. Additionally, studies have shown that cycloaliphatic epoxy resins have the advantages of good weather resistance, excellent electrical insulation properties, high heat resistance, and good process performance [11]. However, the drawback was that the production process of the resin was complex and the preparation cost was high, which limited its wide application. More importantly, scholars at home and abroad have found that the modification of bisphenol A epoxy resin by blending alicyclic epoxy resin can significantly improve the weather resistance, arc resistance and high-temperature adhesion of the epoxy system [12,13,14,15,16]. The blending modification of alicyclic epoxy resin is of great significance to reduce the production cost of epoxy resin and improve the comprehensive properties of epoxy resin. Consequently, it is feasible to enhance the weather resistance of bisphenol A resin by blending alicyclic resin.

The core rods used in composite insulators presently have flaws such as poor weather resistance and easy aging in harsh environments such as high humidity and salt. It is critical to modify the matrix resin used in composite insulating core rods to improve the weather resistance of composite insulators in order to popularize and apply composite insulators in harsh environments such as high humidity and high salt. Herein, alicyclic epoxy resin was selected as the modified resin, and the corrosion aging test of the blend resin system was carried out with 3.5% sodium chloride solution to simulate the accelerated aging process of coastal natural environment in this work. Fourier transform infrared spectroscopy (FTIR), dynamic mechanical analysis (DMA), dielectric loss, leakage current and breakdown field strength of the samples before and after aging were measured and analyzed to explore the weather resistance and aging mechanism of alicyclic blend modified epoxy resin. The results could provide data support and theoretical guidance for the development of weather resistant composite insulation mandrel.

## 2. Materials and Methods

### 2.1. Materials

Bisphenol A epoxy resin (DGEBA) with epoxy value of 0.51 mol/100 g was provided by Zhejiang Pelim Electric Technology Co. (Quzhou, China), Ltd. 3,4-epoxycyclohexylmethyl 3,4-epoxycyclohexylformate (model: 2021P) which was selected as the representative of alicyclic epoxy resin was supplied by Daicel Chemical Industries, Ltd. (Osaka/Japan), and the epoxy value was 0.7~0.75 mol/100 g. 2-methylhexahydrophthalic anhydride (MHHPA) was selected as the curing agent which was supplied by Zhejiang Pelim Electric Technology Co., Ltd.; Tris (Dimethylaminomethyl) phenol (DMP-30) was obtained from Zhejiang Pelim Electric Technology Co., Ltd. as epoxy curing accelerator.

### 2.2. The Preparation of Samples

Blending DGEBA and 2021P, using MHHPA as the curing agent, and changing the proportion of curing agent according to the epoxy equivalent in each proportion of the resin system can improve the curing effect of the epoxy resin system, which can be expressed by Equation (1) [17].

(1) $$\omega (\mathrm{Anhydride})\%=c\times \frac{\mathrm{Anhydride}\;\mathrm{equivalent}}{\mathrm{Epoxy}\;\mathrm{equivalent}}\times 100\%$$

**Figure 1:**
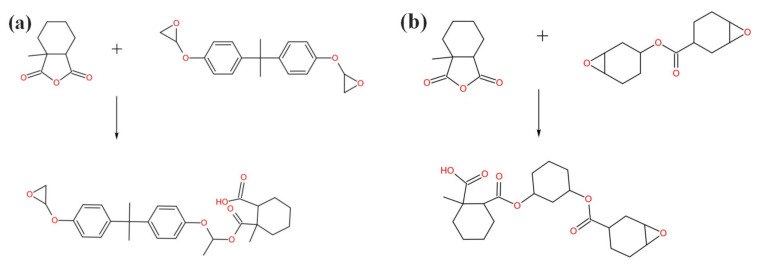
Schematic diagram of curing reaction (**a**) Curing schematic diagram of DGEBA and MHHPA (**b**) Curing schematic diagram of 2021P and MHHPA.

Wherein, *c* is the correction coefficient, and *ω* is the mass percentage of acid anhydride. The actual addition ratio of each component of the blend system was listed in Table 1. The reagents were mixed according to the different formulas in Table 1, kept at a constant temperature of 60 °C, stirred for 7 min, mixed evenly, and prepared a resin glue. Then, the resin glue solution was placed in a vacuum drying oven and subjected to vacuum defoaming treatment at a temperature of 60 °C for 15 min. It was then slowly poured into a mold, preheated at 60 °C, and degassed under a vacuum for 15 min. Finally, the resin glue solution was heated and cured in an oven, and the curing conditions were as follows: the temperature was 140 °C, and the time was 10 h. A schematic diagram of the curing reaction was shown in Figure 1. The cured products of the blend system in which the alicyclic resin accounts for 0 wt%, 10 wt%, and 20 wt% of the total resin mass were named as 0% 2021P/DGEBA(DGEBA), 10% 2021P/DGEBA, and 20% 2021P/DGEBA, respectively.

### 2.3. Media Aging

The corrosive medium simulates natural seawater with a salinity of 3.5% NaCl solution, and the aging cycles were 7 d, 14 d, 21 d, and 28 d respectively. The dielectric loss tangent, leakage current, breakdown field strength, dynamic mechanical analysis and FTIR of the resin samples before and after soaking were measured.

### 2.4. Performance Test Method

#### 2.4.1. Dielectric Loss

The dielectric loss tangent (tanδ) can effectively characterize the dielectric loss of a dielectric material after an electric field is applied [18]. YG9100 automatic anti-interference precision dielectric loss tester (Shanghai Yanggao Electric Co., Ltd., Shanghai, China) was employed to measure the dielectric loss factor. The diameter of the sample was 70 mm, the thickness was 3 mm, and the temperature was 25 °C.

#### 2.4.2. Leakage Current Test

The leakage current of the tested object was measured according to the requirements of the IEC 62217-2012 standard. The height of the sample was 30 mm, the cross-sectional area was 100 mm^2^, the voltage was boosted to 12 kV at a constant rate of 1 kV/s, and the leakage current was measured by digital multimeter (DM-3068, Puyuan Jingdian Technology Co., Ltd., Beijing, China), the current accuracy was ±0.001 μA, and the sampling frequency was 20 Hz.

#### 2.4.3. Power Frequency Breakdown

The breakdown voltage of the test sample were measured according to the GB/T1408.1-2016 standard. Electrodes and samples were immersed in silicone oil to prevent flashover along the surface. The thickness of the sample was 1.0 ± 0.1 mm, the boosting rate was 2 kV/s, at least 20 samples were tested for each sample, and the breakdown field strength of the medium was calculated by the Weibull distribution [19].

#### 2.4.4. FTIR Analysis

Fourier transform infrared spectroscopy (FTIR) was carried out on the sample using IRPrestige-21 Fourier transform infrared spectrometer (Shimadzu Corporation, Kyoto, Japan), the scanning range was 400~4000 cm^−1^, the resolution was 4 cm^−1^, and the number of scans was 32 times.

#### 2.4.5. Dynamic Mechanical Analysis

The single cantilever method of the American TA-Q800 dynamic mechanical analyzer was used to measure the dynamic mechanical properties of the samples. The test frequency was 2 Hz, the test temperature range was 30~200 °C, the heating rate was 5 °C/min, and the test sample size was 35 mm × 10 mm × 1 mm.

#### 2.4.6. Thermogravimetric Analysis

The thermal decomposition temperature and rate were measured by TGA instrument (PerkinElmer-STA6000, Waltham, MA, USA). The test atmosphere is nitrogen, the test vessel is alumina crucible, the test temperature range is 30–800 °C, the heating rate is 10 °C/min, and the sample weight is 5~10 mg.

## 3. Results and Discussion

### 3.1. Analysis of the Influence of Salt Aging on Electrical Properties

#### 3.1.1. Dielectric Loss

**Figure 2:**
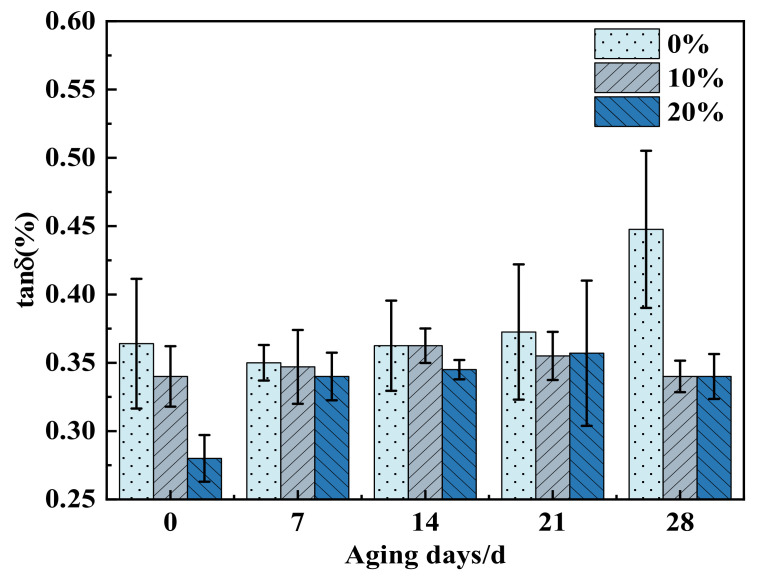
Dielectric loss tangent of cycloaliphatic blend resins with different proportions during salt aging.

Figure 2 displayed the dielectric loss of three proportions of cycloaliphatic blend resins at different aging times. It can be observed from Figure 2 that with the prolongation of aging time, the dielectric loss of DGEBA increases significantly, and the dielectric loss of 10% 2021P/DGEBA and 20% 2021P/DGEBA only increased slightly and remained stable in the later stage of aging. When the aging time was 28 d, the dielectric loss tangent value of 0% 2021P/DGEBA was 0.448%, and the dielectric loss tangent value of 10% and 20% alicyclic blend systems were both 0.34%, which was 31.67% lower than that of 0% 2021P/DGEBA. The reason that the dielectric loss tangent of 0% 2021P/DGEBA increased significantly with the aging time was that Na^+^, Cl^−^ and water molecules in the salt solution environment invade the molecular chain to act as fillers, and the presence of ions will increase the conductivity loss. Meanwhile, the dielectric erosion further expands the defects of the resin material, and interface polarization might occur on the internal impurities and defect interfaces. 10% 2021P/DGEBA and 20% 2021P/DGEBA have no obvious change in the tangent value of dielectric loss under the aging time of 28 d, indicating that the alicyclic blend system has a certain ability to resist medium erosion, with less defects and impurities, and the orientation polarization and conductivity loss of water molecules were small, so good dielectric properties could still be maintained after dielectric aging.

#### 3.1.2. Leakage Current

**Figure 3:**
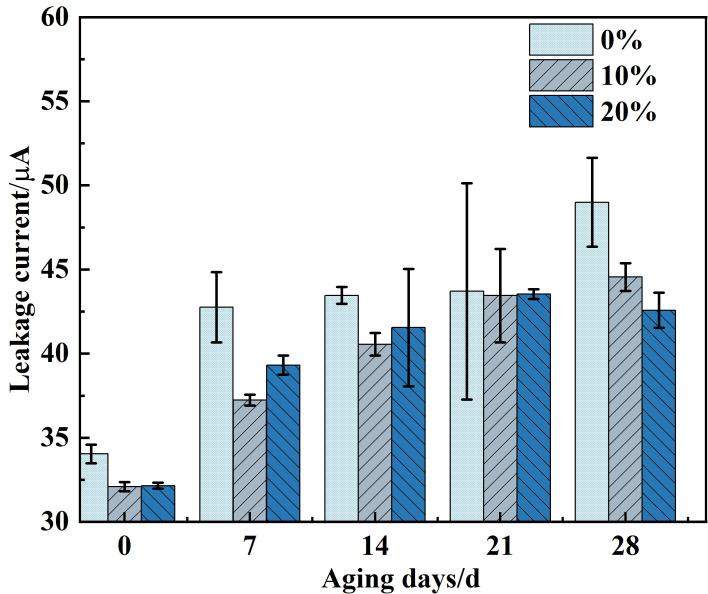
Leakage current of cycloaliphatic blend resins with different proportions during salt aging.

Figure 3 showed the total leakage current curve of alicyclic blends with different proportions under high salt medium aging. With the increase of aging days, the leakage current exhibited an increasing trend. In the aging range of 0~21 d, the leakage current of each resin system showed a slow increasing trend. After aging to 28 d, the leakage current of 0% 2021P/DGEBA increased rapidly to 48.994 μA, while the leakage current value of 10% alicyclic blends was 44.549 μA and tends to be stable, which was 9.07% lower than that of DGEBA, and 20% alicyclic blends were 13.1% lower than that of DGEBA. The analysis results demonstrated that moisture and deterioration will greatly increase the leakage current of insulating medium. The corrosion of salt medium will expand the defects of DGEBA material, and ions and water molecules in high-salt environment will invade the molecular chain, reducing the insulation resistance of the resin, thereby increasing the leakage current. The leakage current of the blend system was always less than that of DGEBA, indicating that the addition of 2021P could reduce the degree of moisture deterioration of the resin system.

#### 3.1.3. Breakdown Field Strength

**Figure 4:**
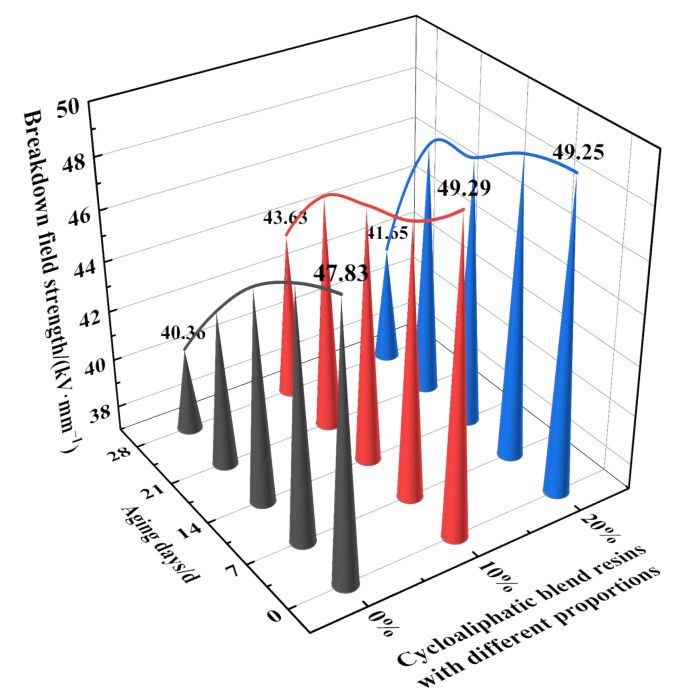
Breakdown field strength of cycloaliphatic blend resins with different proportions during salt aging.

The breakdown field strength of samples prepared by blending 2021P with DGEBA in different proportions under different aging days was calculated by Weibull distribution. Figure 4 depicted the breakdown field strength curves of different proportions of 2021P and DGEBA blends under high-salt medium aging. It can be seen from Figure 4 that the breakdown field strength of the unaged 2021P/DGEBA blended resin system was higher than that of DGEBA, which indicated that the introduction of 2021P molecules could reduce the internal defects of the blended resin system to a certain extent, making it difficult to crack and strike. With the increase of aging days, the breakdown field strength decreased significantly. When the aging days were 28 days, the breakdown field strength of 0% 2021P/DGEBA decreased to 40.35 kV/mm; The breakdown field strength of 10% 2021P/DGEBA was 43.63 kV/mm, which was 8.13% higher than DGEBA; The breakdown field strength of 20% 2021P/DGEBA was 41.65 kV/mm, which was 3.22% higher than that of 0% 2021P/DGEBA. The above reasons were mainly due to the fact that the thickness of the sample was only about 1 mm, which was easy to be corroded in the salt solution. In the process of salt medium erosion, the 0% 2021P/DGEBA sample was seriously damped and degraded, resulting in more uneven distribution of electric field, which will form a discharge channel along the defects at the weakest part of the insulation. The breakdown field strength results of 10% wt epoxy modified tung oil resin prepared by Cao [20] aged for 0 d and 7 d in 3.5% NaCl solution were 56 kv/mm and 39 kv/mm respectively. However, the 10% 2021P/DGEBA can still maintain 43.63 kV/mm after 28 days of salt aging in this paper. It can be seen that the alicyclic blend resin system has fewer defects after salt aging, and the diffusion of ions and water molecules into the resin is inhibited. Therefore, the insulation strength loss of alicyclic blend resin system, especially 10% 2021P/DGEBA, is less.

Based on the above analysis, it can be seen that the high salt medium environment promotes the rapid expansion of the defects of 0% 2021P/DGEBA samples, a large number of Na^+^, Cl^−^ and water molecules in the solution invade the resin, the conductive current of the resin medium also increases, and the insulation performance of DGEBA deteriorates seriously with aging. However, the dielectric and withstand voltage properties of 2021P/DGEBA blend resin, especially 10% 2021P/DGEBA, have been significantly improved compared with DGEBA, which also means that 2021P/DGEBA blend resin has strong insulation and salt corrosion resistance.

### 3.2. FTIR Analysis

The spectra of two crosslinking systems of alicyclic epoxy resin and bisphenol A epoxy resin were measured by FTIR, and the spectra of the two resins were compared. The spectral bands of FTIR characteristic peaks corresponding to main groups were listed in Table 2. It can be seen from Table 2 that the two epoxy resins mainly contain epoxy groups, hydrocarbon groups and other groups, which have characteristic absorption in the mid infrared band. The most significant difference is that DGEBA-MHHPA contains benzene ring, while 2021P-MHHPA contains saturated six membered ring structure (Figure 1).

**Figure 5:**
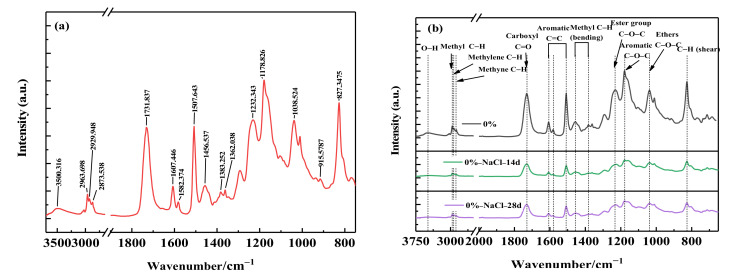

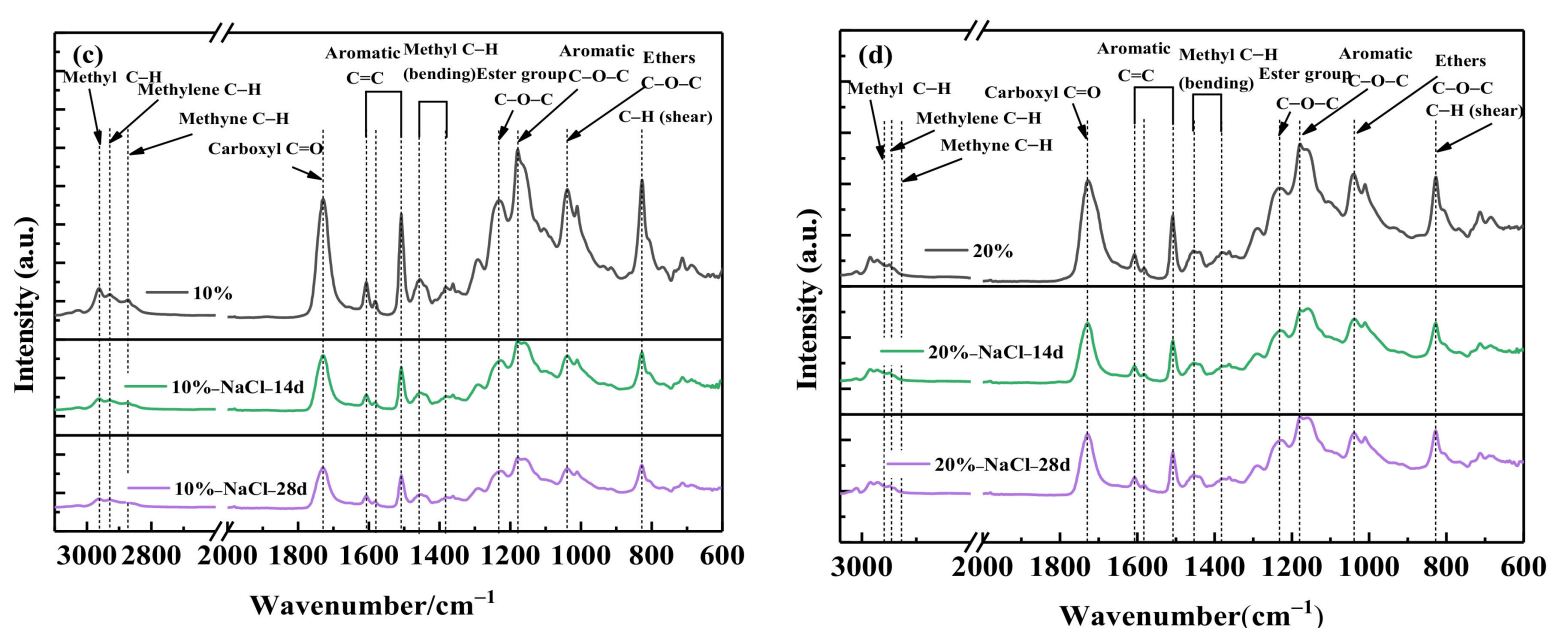
Infrared spectrum of (**a**) 10% 2021P/DGEBA, (**b**) 0% 2021P/DGEBA before and after aging, (**c**) 10% 2021P/DGEBA before and after aging, (**d**) 20% 2021P/DGEBA before and after aging.

Figure 5a showed the FTIR spectrum of 2021P. The 3500 cm^−1^ characteristic peak was attributed to the -OH stretching vibration generated by the crosslinking reaction [21], the 2870–2963 cm^−1^ characteristic peak was assigned to the C-H stretching vibration [22], and 1731 cm^−1^ was the C=O stretching vibration in the molecular structure of 2021P and MHHPA characteristic peaks, the peaks at 1507, 1582 and 1607 cm^−1^ were assigned to benzene ring skeleton vibration, which represent the existence of benzene ring structure in DGEBA, and the peak at 1362 cm^−1^ represents the existence of -C(CH_3_)_2_- structure in DGEBA [19]. 

The FTIR spectra of the composites obtained by blending resins with different proportions after salt medium aging for 0 d, 14 d and 28 d were shown in Figure 5b–d. As can be seen from Figure 5b, in the aging range from 0 to 14 days, the intensity of multiple characteristic peaks such as 1731 cm^−1^, 1507 cm^−1^ and 1178 cm^−1^ for DGEBA decreased significantly [23], suggesting that some functional groups such as hydroxyl, benzene ring skeleton, ester group and aromatic ether in the resin were decomposed [24]. Additionally, it can also be seen that when the aging time was from 14 d to 28 d, the decline of each absorption peak was very small, which also indicated that the chemical structure of the resin has changed significantly in the middle of aging and has been seriously corroded. Further, as can be seen from Figure 5c, with the increase of aging time, the absorption peak intensity of main functional groups in 10% 2021P/DGEBA decreased, but the decrease range was slight. This also demonstrated that adding alicyclic epoxy resin can reduce the corrosion degree of bisphenol A epoxy resin in salt medium. From Figure 5d, the absorption peak intensity of 20% 2021P/DGEBA did not decrease significantly with the increase of aging time in a high-salt medium environment. Thus, it can also be deduced that the chemical structure of cycloaliphatic epoxy resins can indeed remain stable in high-salt environment.

To sum up, comparing the infrared spectra of 0% 2021P/DGEBA, 10% 2021P/DGEBA, and 20% 2021P/DGEBA, it can be concluded that the infrared absorption peaks of 10% 2021P/DGEBA and 20% 2021P/DGEBA only change slightly in the high-salt medium environment. The molecular structure of the resin did not contain a benzene ring and has good chemical stability [23,24]. On the other hand, the epoxy group in 2021P molecular structure did not come from propylene oxide. The epoxy group was directly connected to the alicyclic ring and can form a tight rigid molecular structure. The blending of 2021P and DGEBA resin changes the molecular arrangement and structure of a single resin during curing, and introduces atoms with excellent properties to cross-link with active groups in the resin to improve the structure of matrix DGEBA, and the composite 2021P/DGEBA formed by blending was conducive to jointly resist the inward penetration and diffusion of the medium, so as to improve the corrosion resistance of 2021P/DGEBA, the principle of which was similar to the additive and complementary principle of composite materials [25]. The experimental results also found that the 2021P/DGEBA composite did not suffer from severe corrosion like DGEBA, which fully proved that the alicyclic resin blending method was significantly effective in enhancing the chemical stability of the resin matrix in high-salt media.

### 3.3. Dynamic Mechanical Analysis (DMA)

DMA is a simple method used to measure various transformations of polymer materials and evaluate the heat resistance, cold resistance, compatibility, damping properties and aging properties of materials [26,27,28,29]. Storage modulus represents the storage capacity of energy in the sample under stress, which reflects the elastic component of viscoelastic materials and also reflects the rigidity of the material. The loss factor (tan δ) represents the internal friction of the material and is the tangent of the stress-strain phase angle. Since tan δ is related to molecular relaxation and phase transition, the peak temperature of tan δ in this study is the glass transition temperature (Tg) of the material. To this end, the changing trends of the main parameters storage modulus and loss factor with temperature rise were further explored.

**Figure 6:**
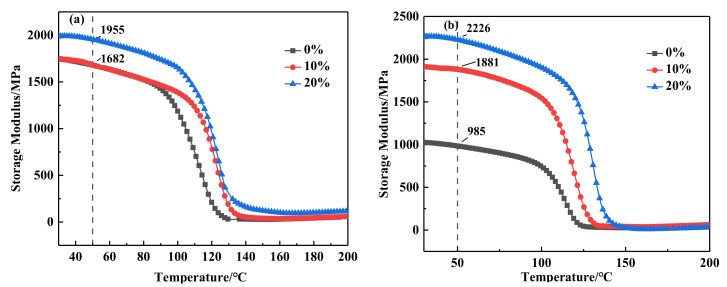
Storage modulus-temperature spectrum of each resin system under different aging conditions (**a**) Stoage modulus of cycloaliphatic blend resins with different proportions without aging (**b**) Storage modulus of cycloaliphatic blend resins with different proportions after 28 days salt aging.

Figure 6 displayed the change of storage modulus with temperature of alicyclic epoxy resin and DGEBA blends in different proportions under different aging time. As can be seen from Figure 6a, for the three non aged blends, the storage modulus increases with the increase of the addition proportion of alicyclic epoxy resin. When the content of alicyclic epoxy resin was 20%, the storage modulus of 20% 2021P/DGEBA was much higher than 0% 2021P/DGEBA, reaching 1955 MPa. According to the molecular structure of 2021P, the epoxy group in the molecule is directly connected with the saturated six membered ring, and the molecular chain rigidity was large. The introduction of 2021P epoxy resin in an appropriate amount can enhance the molecular chain rigidity and storage modulus of 2021P/DGEBA composites. Figure 6b showed the change curve of the storage modulus of the three blends after 28 days of salt aging. After salt aging, the storage modulus of 0% 2021P/DGEBA decreased significantly, and the storage modulus of 10% 2021P/DGEBA and 20% 2021P/DGEBA was much higher than that of 0% 2021P/DGEBA, reaching 2226 MPa and 1881 MPa respectively, exceeding that of 0% 2021P/DGEBA, 1241 MPa and 895 MPa. Lu [30] discovered that the storage modulus of epoxy resin in salt spray environment is close to that before aging, which is about 850 MPa. The storage modulus of 0% 2021P/DGEBA decreases significantly in this paper, and the storage modulus of 10% 2021P/DGEBA and 20% 2021P/DGEBA is close to that before aging. The reason for the above phenomenon was that with the aging, DGEBA was seriously hydrolyzed and all functional groups were decomposed and destroyed; Moreover, a large number of defects were generated on the surface, and water molecules invade the interior of the resin, which played a plasticizing role for many polar functional groups of the resin, resulting in a decrease in the storage modulus [31]. The mechanical modulus of the blend material was not significantly improved after 2021P resin aging, because the molecular energy storage modulus of the blend material was still slightly improved before and after 2021P resin aging, which indicated that the storage modulus of the blend material was still slightly improved, and the molecular energy storage modulus of the blend material was not even improved after 2021P resin aging.

**Figure 7:**
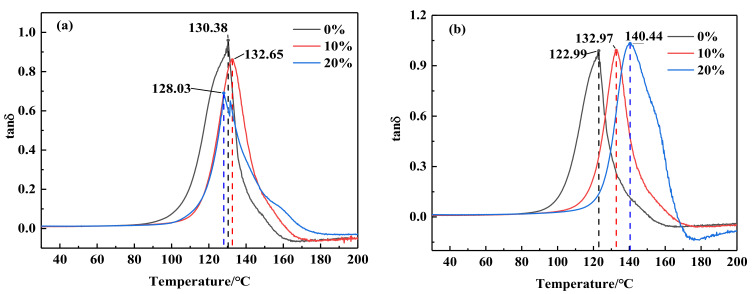
Loss factor −temperature spectrum of each resin system under different aging conditions (**a**)Loss factor without aging (**b**)Loss factor after 28 d salt aging.

The loss coefficient-temperature curve can reflect the compatibility of the two blended resin polymers, and the blending modification is an effective method to improve the physical properties of the material to meet different needs. If the two resins are completely compatible, the properties of the blended resin are almost identical to that of a random copolymer with the same composition. If the compatibility is poor, the blended system will form two independent phases, the two phases show different properties of the two components, and the loss coefficient-temperature curve will show two loss peaks. If the two components have a certain degree of compatibility, the Tg of the two components will be close to each other. Figure 7a depicted the loss coefficient-temperature diagram of the three blend systems when they were not aged. The Tg of the three samples was analyzed. When 10% proportion of 2021P cycloaliphatic resin was added, the loss temperature curve has only one peak, indicating that the two were fully compatible. Although there are two peaks in the complex formed by blending 20% 2021P and DGEBA, the two peaks were very close and highly similar, which showed that the compatibility between the two was still very good and almost completely compatible. More, it can be seen from Figure 7a that the Tg of 0% 2021P/DGEBA was 130.38 °C, and as the proportion of 2021P epoxy resin increased, the Tg showed a trend of first rising and then falling. When the proportion of 2021P epoxy resin increased to 10%, the Tg increased to 132.65 °C, and when the proportion of 2021P epoxy resin reached 20%, the Tg decreased to 128.03 °C. The reason for this phenomenon was mainly due to the fact that the molecular structure of 2021P epoxy resin was more compact than that of DGEBA, and the introduction of an appropriate amount of 2021P epoxy resin can increase the epoxy content and crosslinking density of the 2021P/DGEBA composite, so that the molecular weight of the composite can be increased. The chain degrees of freedom decrease, eventually leading to an increase in Tg. However, when the content of 2021P exceeded the critical value, because the structure of 2021P molecules aggregates, it is not easy to cross-link to form long chains, so the degree of freedom of the molecular chains of the blend system increases, resulting in a decrease in the glass transition temperature. 

Figure 7b showed the loss coefficient temperature diagram of three blends aged in salt medium. It can be seen from the figure that after 28 days of salt aging, the loss modulus and storage modulus of 2021P and DGEBA blend composites with different proportions were much higher than those of DGEBA. The specific performance was as follows: the loss temperature curves of the three blends with salt aging have only one peak, and the Tg value of 20% 2021P/DGEBA was the largest, reaching 140.44 °C, followed by 10% 2021P/DGEBA, the Tg value was 132.97 °C. The Tg value of 0% 2021P/DGEBA was greatly reduced, which was 122.99 °C. Yoon et al. [32] discovered that the Tg of carbon/epoxy resin composites in 5% NaCl saline environment for one month was 95 °C, which decreased by 10.3 °C compared with that before aging, similar to the Tg change of DGEBA in saline environment in this paper. The test phenomenon in this paper was that after 28 days of aging in salt medium, the molecular chain of DGEBA was relaxed and most of the molecular chains of functional groups were broken, resulting in the increase of molecular free volume [33]. Additionally, water entering the resin increases the distance between molecular chains and weakens the interaction between molecular chains, enhanced the motion ability of molecular chains and decreased Tg. For 2021P/DGEBA blend system, 2021P epoxy resin was not easy to be eroded by medium aging, the degree of hydrolysis of resin structure was small, and a small amount of water molecules invade the interior of the resin, which might form hydrogen bonds with the molecular chains of the resin matrix, causing secondary crosslinking between molecular chains and improving the crosslinking density of the matrix. The hydrogen bond formed by water molecules and metal ion Na^+^ could weaken the activity of molecular chain and increase Tg. Obviously, 2021P/DGEBA blend epoxy resin system possessed excellent salt aging resistance.

### 3.4. Thermogravimetric Analysis

**Figure 8:**
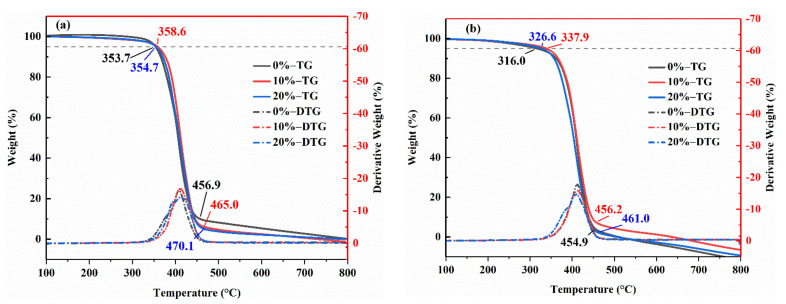
TG and DTG curves of various resin systems under different aging conditions (**a**) Without aging (**b**) After salt aging.

The thermal properties of the resin can be characterized by the initial decomposition temperature (T_i_). Generally, the higher the T_i_, the better the heat resistance of the material. The initial decomposition temperature is defined as the temperature at which the material loses 5% of its weight. Figure 8a showed the TG and DTG curves of the three blends without aging. It can be seen from the figure that the T_i_ of 10% 2021P/DGEBA and 20% 2021P/DGEBA are 358.6 °C and 354.7 °C, respectively. The Ti of the alicyclic blend modification system is higher than that of 0% 2021P/DGEBA. Meanwhile, the final decomposition temperature (T_f_) of 10% 2021P/DGEBA and 20% 2021P/DGEBA are 465.0 °C and 470.1 °C, respectively, which are also higher than 0% 2021P/DGEBA. The main reason for this change is that the alicyclic modified epoxy resin has a high crosslinking density after curing, and the introduction of an appropriate amount of 2021P molecules can improve the thermal stability of the resin system.

Figure 8b showed the TG and DTG curves of the three blends during salt aging. The T_i_ of 0% 2021P/DGEBA, 10% 2021P/DGEBA, and 20% 2021P/DGEBA declined by 37.7 °C, 20.7 °C, and 28.1 °C, respectively, after salt aging. DGEBA’s T_i_ decline was severe, but the T_i_ declines of 10% 2021P/DGEBA and 20% 2021P/DGEBA were significantly lower than DGEBA’s, and the T_f_ was still higher than DGEBA’s. The thermal stability of 10% 2021P/DGEBA before and after aging was the best. It showed that adding an appropriate amount of 2021P molecular modified epoxy resin can not only improve the degree of crosslinking and increase the thermal stability of the resin, but also improve the molecular structure and arrangement of the resin, increasing salt corrosion resistance and maintaining stable thermal properties. Thermal investigation revealed that the alicyclic blended modified epoxy resin has a high level of salt corrosion resistance.

According to the above analysis, 2021P/DGEBA composites were prepared by blending 2021P epoxy resin and DGEBA. The insulation performance of the composite was closely related to the degree of moisture and deterioration. In the environment of high salt medium, the matrix resin DGEBA was seriously hydrolyzed. With the expansion of defects, a large number of Na^+^, Cl^−^ and water molecules in the solution invade the resin, the conductive current of the resin medium also increases, and the insulation performance deteriorates. On the contrary, 2021P/DGEBA composites possessed high stability during salt medium aging. This is mainly due to the formation of flexible long chains during the curing of DGEBA-MHHPA, while the molecular chains in the alicyclic epoxy resin 2021P can form a tight rigid structure. The blending mode of 2021P epoxy resin and DGEBA changed the molecular arrangement and structure during the curing of DGEBA. 2021P epoxy resin with excellent weather resistance was introduced to improve the stability of DGEBA molecular structure, which was conducive to jointly resist the penetration and diffusion of external media into the material and reduce the aging defects of the resin matrix itself, which prevented the deep invasion of Na^+^, Cl^−^ and water molecules to a certain extent, and greatly improves the insulation and salt corrosion resistance of 2021P/DGEBA blend composites.

## 4. Conclusions

In this work, three kinds of alicyclic epoxy/bisphenol A epoxy resin composites with different proportions (0% 2021P/DGEBA, 10% 2021P/DGEBA, 20% 2021P/DGEBA) were prepared by facile blending modification method. In the high salt medium environment, the weather resistance of these composites was measured and analyzed by means of infrared spectroscopy, dynamic mechanical properties, thermogravimetric analysis, dielectric properties, leakage current, breakdown field strength, and other test methods. Finally, the best weather-resistant materials were selected, and their enhanced aging mechanism was discussed. DGEBA-MHHPA formed a flexible long chain during curing, while the molecular chain of alicyclic epoxy resin 2021P could form a tight rigid structure. The blending mode of 2021P epoxy resin and DGEBA changed the molecular arrangement and structure during curing. The introduction of 2021P epoxy resin with excellent weather resistance enhanced the stability of DGEBA molecular structure, hinders the penetration, diffusion, and erosion of external media (such as Na^+^, Cl^−^, water molecules, etc.) into the material to a great extent, reduced the aging defects of the resin matrix itself, and promoted the insulation and salt corrosion resistance of 2021P/DGEBA blend composites to be greatly improved. Based on the excellent weather resistance and electrical insulation performance of 2021P/DGEBA, and comprehensively considering the cost of the material, 10% 2021P/DGEBA blend resin material was selected as the core rods matrix material of composite insulator, composite cross arm and other harsh weather environments, which has broad application prospects.

## Figures and Tables

**Table 1:** Addition proportion of components in different blends.

Samples	DGEBA (g)	2021P (g)	MHHPA (g)	DMP-30 (g)
0% 2021P/DGEBA	100	0	75	0.525
10% 2021P/DGEBA	90	10	77.5	0.533
20% 2021P/DGEBA	80	20	80	0.54

**Table 2:** Spectral bands of FTIR characteristic peaks corresponding to main groups.

IR Bands (cm^−1^)	Assignment
3500	O−H stretching
2963	Stretching C−H of methyl
2926	Stretching C−H of methylene
2870	Stretching C−H of methyne
1730	Carboxy C=O
1608, 1581, 1509	Stretching C=C of aromatic rings
1459, 1383	Bending C−H of methyl
1361	Deformation CH_3_ of C− (CH_3_)_2_
1280~1100	Stretching C−O−C of esters
1250~1190	Stretching C−C of alkanes
1181	Stretching C−O−C of aromatic
1036	Stretching C−O−C of ethers
915	Stretching C−O of oxirane group
827	Shear C−H

## Data Availability

The data presented in this study are available on request from the first authors and corresponding author.
